# Totally endoscopic ventricular septal defect repair using bilateral femoral arterial cannulation in an 8-year-old girl

**DOI:** 10.1016/j.ijscr.2018.12.002

**Published:** 2019-01-09

**Authors:** Huy Q. Dang, Huong T. Le

**Affiliations:** a Minimally Invasive Cardiac Surgery Unit, Cardiovascular Center, Hanoi Heart Hospital, Hanoi, Viet Nam; b Institute of Preventive Medicine and Public Health, Hanoi Medical University (HMU), Hanoi, Viet Nam

**Keywords:** Ventricular septal defect, Femoral arterial cannulation, Totally endoscopic surgery, Robotic surgery

## Abstract

**Introduction:**

There are few reports on applying totally endoscopic surgery (TES) for repairing ventricular septal defect (VSD), especially in children.

**Case presentation:**

We described an 8-year-old girl, weighing 17 kg, who was diagnosed with peri-membranous VSD and patent ductus arteriosus (PDA). The patient had undergone trans-catheter PDA closure first, and 3 weeks later, TES was performed for VSD repair through 4 small trocars (one 12 mm trocar and three 5 mm trocars) without robotic assistance. Arterial line was set up indirectly to the right femoral artery (FA) through a graft with an additional line to the left side for reducing arterial pressure.

**Discussion:**

FA cannulation in small children induce some risks, such as: FA trauma, critical lower limb ischaemia, and iliac or femoral arterial stenosis. Our new method of FA cannulation was safe with 13 months of follow-up. The major concerns in this case were repairing VSD through small trocars in a small child.

**Conclusion:**

Bilateral FA cannulation and the way to set up small trocars may facilitate totally endoscopic VSD repair in small children. However, the safety and efficacy of these approaches needs to be validated by larger studies preferably randomised controlled trials.

## Highlights

• The scope of totally endoscopic cardiac surgery in children is limited.
• Femoral cannulation has risks which increase in small children.
• Bilateral femoral arterial cannulation helped to avoid vessel complication in small children.
• Four small trocars (5–12 mm) were suitable for totally endoscopic VSD closure.

## 1 Introduction

Ventricular septal defect (VSD) is one of the most common congenital heart diseases with the prevalence of 30% [1]. Although minimally invasive cardiac surgery – MICS – has evolved rapidly in recent years, its application in VSD closure is still limited. Until now, there are few reports on totally endoscopic surgery (TES) for the treatment of VSD, especially in small children [[2], [3], [4]]. In this report, we used new methods to set up FA cannulae and small trocars for closing VSD in an 8-year-old female patient, without robotic assistance. The work has been reported in accordance with the SCARE criteria [5].

## 2 Case report

An 8-year-old female patient, weighed 17 kg had been diagnosed with congenital heart disease during her infancy, with unknown follow-up and treatment. She was admitted to the hospital due to fatigue, shortness of breath while playing with friends one month ago. Physical examination on admission revealed a systolic murmur in the left para-sternum, trans-thoracic echocardiography showed a peri-membranous VSD extended into inlet septum with a diameter of 12 mm, left to right shunting, pressure gradient via the defect was 20 mmHg, a PDA of 4 mm in diameter. The patient had undergone trans-catheter PDA closure first, and 3 weeks later, TES was performed for VSD repair without robotic assistance.

Patient was placed in supine position; two arms were along the body, under general anesthesia with single-lumen endotracheal tube. We used a no.6 Knitted Dacron graft (Vascutek Terumo, Bangkok, Thailand) which was connected to the right common FA of the patient with an end-to-side anastomosis. The other side of the graft was connected to the arterial line of cardiopulmonary bypass (CPB) machine. Superior vena cava (SVC) and inferior vena cava (IVC) cannulae were inserted via internal jugular vein (IJV) and femoral vein, respectively, using Seldinger technique. When CPB was started, the arterial pressure gradually increased to 260 mmHg, we switched to bilateral FA cannulation with an additional 10Fr FA cannula (Medtronic, Inc., Minneapolis, Minn, USA) (the predicted size was 16Fr) was directly inserted to the left common FA. After having additional femoral cannula, the arterial pressure declined and stabilized at 180–200 mmHg.

Four soft trocars (Covidien, Hampshire, Mansfield, USA) were placed in the right chest of the patient, included: one 12 mm trocar in the 5^th^ intercostal space (ICS) at the anterior axillary line as the main working port, one 5 mm trocar in the 4^th^ ICS at the mid-axillary line as the second working port, one 5 mm trocar in the 5^th^ ICS at mid-axillary line as the camera port and one 5 mm trocar in the 6^th^ ICS at the mid-axillary line for left atrium sucker. After snaring SVC, we pushed a 16 G Surflo^®^ I.V. Catheters (Terumo Corporation, Laguna, Philippines) into the ascending aorta through the anterior right chest wall and used it as an aortic root needle. Myocardial protection was achieved by antegrade Custodiol^®^ HTK solution (Koehler Chemi, Alsbach-Haenlien, Germany) through aortic root after clamping aorta by Chitwood**^®^** clamp (Scanlan International, Inc, St Paul, MN, USA).

**Fig. 1:**
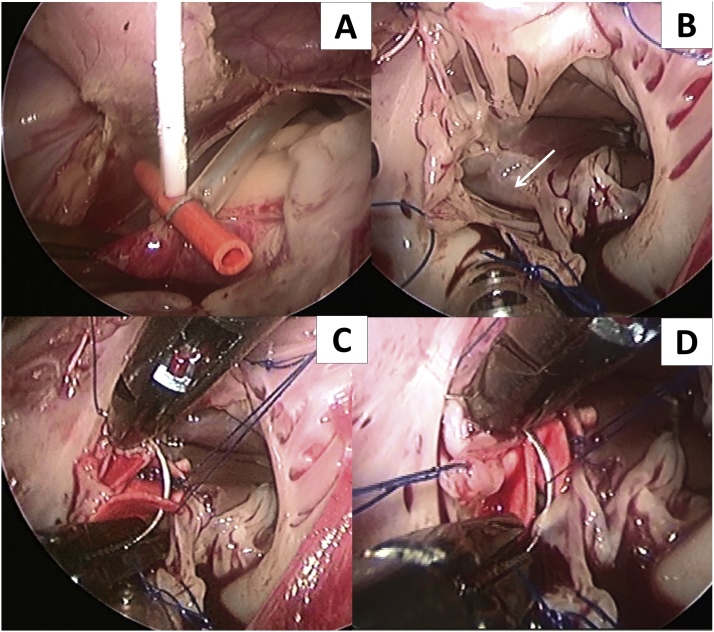
(A): Aorta cross-clamp by Chitwood clamp, antegrade cardioplegia using Custodiol HTK solution via aortic root with aorta needle via chest wall puncture; (B): Peri-membranous VSD extended into inlet septum (white arrow), many tendons crossed the upper and lower margin of the defect; (C, D): VSD closure using artificial patch, continuous combined with interrupted suture.

**Fig. 2:**
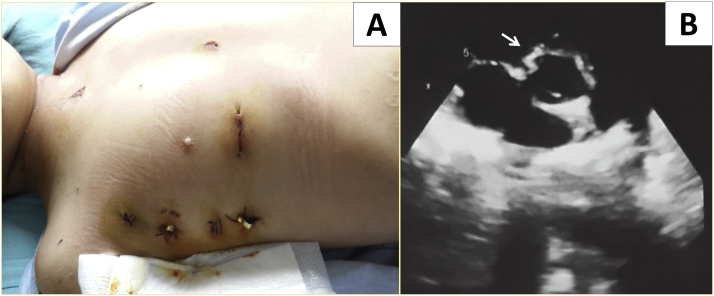
(A): Surgical scars prior to discharge; (B): Artificial patch (white arrow) in parasternal short axis view.

Right atrium (RA) was opened in parallel to atrioventricular groove, atrial septal was intact, and we punctured atrial septum at the location of ovale foramen to place the drainage of left atrium. The defect was exposed by taking some stitches to hang RA wall to the pericardium. The lesion extended into the inlet septum, some chordae tendineae of septal and anterior leaflets of tricuspid valve crossed the defect. We split the commissure of anterior and septal leaflets to clearly expose the defect. The VSD was closed using artificial patch, continuous combined with interrupted suture (Fig. 1). The RA was closed using two layer of continuous suture after the split in the leaflets and the ovale foramen had been closed. After declamping the aorta, the heart re-beat in sinus rhythm, de-airing via aortic root needle. Extracorporeal circulation was then stopped and the operation was finished with no difficulties. CPB time and aorta cross-clamp (ACC) time were 185 and 150 min, respectively. Echocardiography prior to discharge showed completely closed VSD, no tricuspid regurgitation. The patient was discharged at the postoperative day 8 without any symptoms; no neurological or vascular complications were noted at the follow-up visit 3 months after surgery. Her family was very satisfied with the results (Fig. 2).

## 3 Discussion

TES is rarely used in small children due to risks of peripheral CPB. However direct FA cannulation is the standard technique, it predispose the small patients to some risks: (1) the pressure of the arterial line may increase gradually during the operation due to the reflex arterial spasm, especially in children, (2) acute lower limb ischaemia during and after surgery, and (3) postoperative stenosis of the iliac or femoral arteries [6]. As in our other reports [[7], [8], [9]], we used a Knitted Dacron graft (Vascutek Terumo, Bangkok, Thailand) to connect to the common FA of the patient with an end-to-side anastomosis. At the end of the operation, the graft was cut as near as possible to the anastomosis. The remains of the graft were closed simply. This method completely eliminated the risks of the leg ischaemia and the postoperative arterial stenosis [6].

In case the pressure of the arterial line increased, a FA cannula which was 4-6Fr smaller than the diameter of FA was inserted into left FA to reduce the pressure. After removing this cannula at the end of the operation, the hole on the arterial wall was closed horizontally. This method was not only effective in reducing the pressure of the arterial line (80–100 mmHg) but also avoid the arterial trauma which would induce postoperative stenosis of the iliac or femoral arteries with 13 months of follow-up [7]. We believe that this method is a good resolution for setting up peripheral CPB in small children.

Different from ASD, there are few reports on applying TES for repairing VSD due to risks of AV block or residual VSD. Ma et al [2,3] and Gao et al [4,10] used 3 big size trocars (15–25 mm) so that one or two instruments can put into the thoracic cavity through one of them. In addition two of these three trocars located on the anterior chest wall. Although this approach made manipulation easy and reduced ACC time, the scars seemed to be not cosmetic [2,3]. In this report, we used smaller trocars (5–12 mm) and most of them located in mid-axillary line so that post-operative scars were behind the right arm.

The CPB time and ACC time were long due to difficulty in closing VSD (some chordae tendineae of septal and anterior leaflets of tricuspid valve crossed the defect) and deficiency of learning curve. We think that it would be much more difficult if the lesion was closer to aortic valve. Therefore, this technique should only be applied to carefully selected patients.

## 4 Conclusion

Bilateral FA cannulation and the way to set up small trocars may facilitate totally endoscopic VSD repair in small children. However, the safety and efficacy of these approaches needs to be validated by larger studies preferably randomised controlled trials.

## Conflicts of interest

No conflict of interest declared.

## Sources of funding

No funding was received for the study.

## Ethical approval

Ethical approval is not needed in Vietnam.

## Consent

Written informed consent was obtained from the parents of the patient for publication of this case report and accompanying images. A copy of the written consent is available for review by the Editor-in-Chief of this journal on request.

## Author contribution

All authors: Dr. Dang and dr. Le have taken part in conception of the study, drafting and revising the whole manuscript critically. All authors have given their final approval of the manuscript upon submission.

## Registration of research studies

None.

## Guarantor

Huy Q. Dang.

## Provenance and peer review

Not commissioned, externally peer-reviewed
